# Thrombospondin-1 secreted by human umbilical cord blood-derived mesenchymal stem cells rescues neurons from synaptic dysfunction in Alzheimer’s disease model

**DOI:** 10.1038/s41598-017-18542-0

**Published:** 2018-01-10

**Authors:** Dong Hyun Kim, Hoon Lim, Dahm Lee, Soo Jin Choi, Wonil Oh, Yoon Sun Yang, Jeong Su Oh, Hyun Ho Hwang, Hong Bae Jeon

**Affiliations:** 1 Biomedical Research Institute, R&D Center, MEDIPOST Co., Ltd, Gyeonggi-do, Republic of Korea; 2 Department of Genetic Engineering, College of Biotechnology and Bioengineering, Sungkyunkwan University, Suwon, Republic of Korea; 3 King Abdullah University of Science and Technology, Thuwal, Saudi Arabia

## Abstract

Alzheimer’s disease (AD) is an incurable neurodegenerative disease characterised clinically by learning and memory impairments. Amyloid beta (Aβ) peptide-induced synaptic dysfunction is a pathological process associated with early-stage AD. Here, we show that paracrine action of human umbilical cord blood-derived-mesenchymal stem cells (hUCB-MSCs) protects the hippocampus from synaptic-density loss in *in vitro* and *in vivo* AD models. To identify paracrine factors underlying this rescue effect, we analysed hUCB-MSCs’ secretome co-cultured with Aβ42-treated mouse hippocampal neurons. Thrombospondin-1 (TSP-1), a protein secreted by hUCB-MSCs in *in vitro* and 5XFAD AD mouse models, was selected for study. Treatment with exogenous recombinant TSP-1 or co-cultures with hUCB-MSCs significantly increased expression of synaptic-density markers, such as synaptophysin (SYP) and post-synaptic density protein-95 (PSD-95) in Aβ42-treated mouse hippocampal neurons. Knockdown of TSP-1 expression in hUCB-MSCs through small interfering RNA (siRNA) abolished the reversal of Aβ42-induced hippocampal synaptic-density loss. We demonstrate that the rescue effect of hUCB-MSC-secreted TSP-1 was mediated by neuroligin-1 (NLGN1) or α2δ-1 receptors. Interestingly, NLGN1 and α2δ-1 expression, which was reduced in Aβ42-treated hippocampal neurons, increased in co-cultures with hUCB-MSCs or exogenous TSP-1. Together, these findings suggest that hUCB-MSCs can attenuate Aβ42-induced synaptic dysfunction by regulating TSP-1 release, thus providing a potential alternative therapeutic option for early-stage AD.

## Introduction

Alzheimer’s disease (AD) is the most common and progressive neurodegenerative disease worldwide^1^. The progression of AD is characterised by pathophysiological features such as the accumulation of amyloid beta (Aβ) peptide forming senile plaques in the brain, intracellular neurofibrillary tangles, and synaptic degeneration^2,3^. Severe cognitive impairment and memory loss are the clinical hallmarks of AD^4^. These symptoms are associated with hippocampal synaptic dysfunction caused by the presence of soluble Aβ peptide^5^. Recently, growing evidence has shown that soluble Aβ peptide has toxic effects on synaptic function and plasticity^6^; this supports the occurrence of Aβ peptide-induced synaptic dysfunction in AD, which is an essential pathogenic event in patients with early-stage AD^7–10^. Thus, soluble Aβ peptide is a molecular trigger that accelerates AD pathogenesis^11^. Indeed, soluble Aβ peptide-induced hippocampal synaptic dysfunction, as evidenced by reduced expression of synaptic density markers such as synaptophysin (SYP) and post-synaptic density protein-95 (PSD-95)^12,13^, has been reported in AD patients and AD mouse models^14^. Although finding successful therapies for AD is currently very challenging, resolving synaptic dysfunction in the early stages of AD is an attractive target for therapeutic intervention.

Human umbilical cord blood-derived-mesenchymal stem cells (hUCB-MSCs) have been emerging as an alternative cellular source for allogeneic MSC-based therapy due to their beneficial characteristics, including a noninvasive method for their collection, hypo-immunogenicity, superior tropism, high differentiation potentials, and paracrine activity^15–17^. Based on these biological activities, our previous studies have shown that hUCB-MSCs have therapeutic effects on Aβ peptide-dependent AD pathology. For instance, the secretome of hUCB-MSCs includes therapeutic molecules such as galectin-3, which has an anti-apoptotic effect on neuronal cells^18^, intracellular adhesion molecule-1 (ICAM-1), which clears Aβ peptide plaques^19^, and growth differentiation factor-15 (GDF-15), which promotes neurogenesis in AD models^20^. The therapeutic effects of hUCB-MSCs have also been verified in an AD mouse model^19,20^. Thus, our research group is currently conducting Phase-I/IIa clinical trials for the treatment of AD with the approval of the Korean Food and Drug Administration (Clinical Trials Gov Identifier: NCT02054208). However, although we have found that transplantation of hUCB-MSCs significantly improved cognitive function and memory in the AD mouse model^20^ and expect the same improvements in the AD patients participating in the clinical trials, further research is required to understand the mechanism by which synaptic dysfunction is closely linked to cognitive and memory impairment in AD patients.

In this study, we investigated whether the paracrine action of hUCB-MSCs can rescue neurons from Aβ peptide-induced synaptic dysfunction in AD models. To address this issue, we established an *in vitro* model using a co-culture system of hUCB-MSCs and mouse primary hippocampal neurons treated with a low-dose, soluble Aβ peptide, which induces synaptic damage without causing neuronal cell death. In addition, to determine which paracrine factors in hUCB-MSCs have a rescuing effect from Aβ peptide-induced loss of synaptic density, we analysed the secretome of the co-culture media under the same *in vitro* conditions. One of the proteins upregulated in the media, thrombospondin-1 (TSP-1), was selected, and its rescue effect and mechanism regarding Aβ peptide-induced synaptic dysfunction were further validated.

## Results

### Treatment of hUCB-MSCs rescue hippocampal neurons from Aβ42 peptide-induced loss in synaptic density in *in vitro* and *in vivo* AD models

**Figure 1:**
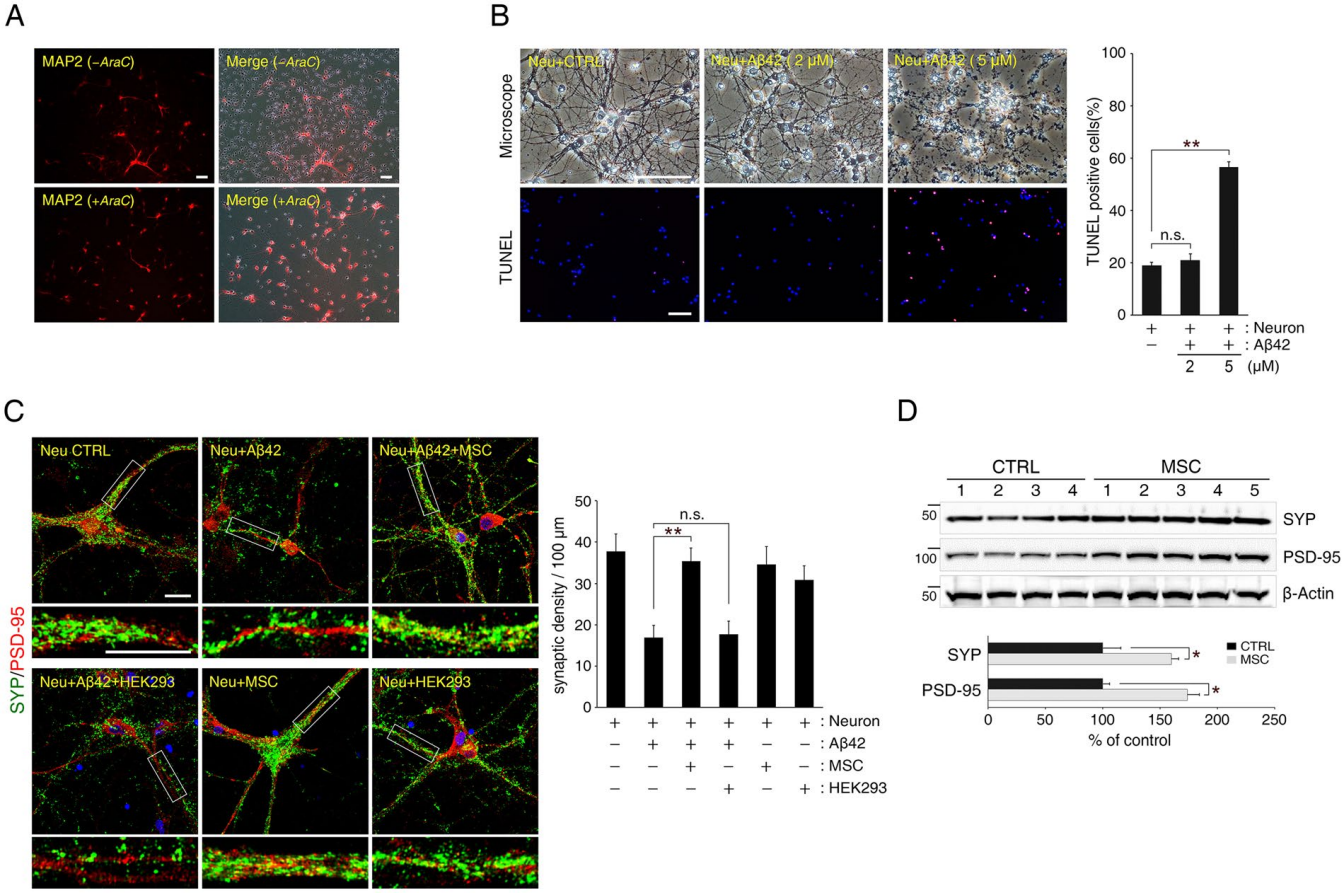
Co-culture with human umbilical cord blood-derived-mesenchymal stem cells (hUCB-MSCs) prevents reduction in synaptic density in Aβ42 peptide-treated primary hippocampal neurons. (**A**) Contamination by non-neuronal cells was removed with cytosine arabinoside (AraC) treatment of the primary hippocampal cell culture, and differentiated cells were confirmed to be mostly hippocampal neurons by staining for microtubule-associated protein 2 (MAP2), a neuron-specific marker (Scale bar = 200 μm). (**B**) Aβ42 peptide-induced cytotoxicity was measured in primary hippocampal neurons at 72 h after treatment in a dose-dependent manner. Treatment with 2 μM of Aβ42 peptide was verified via analysis of microscope images and TUNEL assay, confirming that this concentration did not cause cytotoxicity in hippocampal neurons (Scale bar = 100 μm, mean ± SEM, ** p < 0.005, n = 3 per group, n.s.: not significant). (**C**) Representative images of hippocampal neurons stained with antibodies specific for pre-synaptic (SYP, green) and post-synaptic (PSD-95, red) proteins (Scale bar = 25 μm, Neu: neuron). Bottom insets (white boxes) show higher magnification. Quantification of synaptic density (number of synapses per 100 μm of dendritic length, n ≥ 30 dendrites) revealed that co-culture with hUCB-MSCs rescued hippocampal neurons from Aβ42 peptide-induced synaptic dysfunction (mean ± SEM, **p < 0.005, n.s.: not significant). (**D**) hUCB-MSCs-administered mouse brains were extracted and analysed by immunoblotting for SYP and PSD-95 antibodies. β-Actin was used as a loading control. (n = 4 for CTRL: MEMα-administrated 5XFAD, n = 5 for MSC: hUCB-MSC-administered 5XFAD, *p < 0.05).

To investigate the role of hUCB-MSCs on Aβ peptide-induced synaptic dysfunction in primary hippocampal neurons, we established a co-culture system. First, the culture was treated with cytosine arabinoside (AraC) to exclude effects on non-neuronal cells. This was confirmed by staining for microtubule-associated protein 2 (MAP2), a neuron-specific marker (Fig. 1A). Next, we sought to find the optimal Aβ42 peptide concentration that does not induce neuronal cell death. Based on previous studies^18,21^, we tested the neurotoxicity of concentrations between 2 μM and 5 μM of Aβ42 peptide. After analysing optical microscopy images and a Terminal deoxynucleotidyl transferase dUTP nick end labelling (TUNEL) assay, we found that 2 μM of Aβ42 peptide-treated hippocampal neurons showed no cell death phenotypes as compared to results for the untreated control group. However, 5 μM of Aβ42 peptide-treated hippocampal neurons did reveal increased cell death by neurotoxicity as compared to results for either the untreated control or the group treated with 2 μM of Aβ42 peptide (Fig. 1B). After establishing an ‘*in vitro* AD system*,’* we evaluated the synaptic density of neurons to investigate synaptic function in primary hippocampal neurons.

Synaptic density was assessed as the frequency of co-localisation of the pre- and post-synaptic markers SYP and PSD-95, respectively, in the hippocampal neurons^22^.

We confirmed that Aβ42 peptide induces synaptic dysfunction in primary hippocampal neurons through reduction in synaptic density. However, a co-culture with hUCB-MSCs prevented Aβ peptide-induced synaptic loss in primary hippocampal neurons. Co-cultures with other human-origin cells, such as human embryonic kidney 293 (HEK293) cells, did not rescue neurons from Aβ peptide-induced loss of synaptic density. Co-cultures with hUCB-MSCs or HEK293 cells without Aβ42 peptide treatment had no effect on synaptic density in primary hippocampal neurons (Fig. 1C).

Next, to evaluate whether synaptic dysfunction could be improved by hUCB-MSCs in an AD mouse model, hUCB-MSCs (1 × 10^5^ cells per head) were transplanted via a cannula into the lateral ventricle of 6-month-old 5XFAD mice. On analysing the expression of typical synaptic markers via immunoblotting, at 4 weeks after transplanting hUCB-MSCs into 5XFAD mice, levels of SYP and PSD-95 were significantly upregulated throughout the entire brain (SYP: 1.60-fold increase and PSD-95: 1.74-fold increase; Fig. 1D).

These data show that soluble Aβ peptide can induce loss in synaptic density in hippocampal neurons and that treatment with hUCB-MSCs can rescue the neurons from Aβ peptide-induced synaptic dysfunction in *in vitro* and *in vivo* AD mouse models.

### Identification of TSP-1 as a paracrine factor of hUCB-MSCs in co-culture with primary hippocampal neurons under Aβ42 peptide treatment

Based on the paracrine effect of hUCB-MSCs that prevents Aβ peptide-induced loss in synaptic density, we sought to identify soluble factors derived from hUCB-MSCs. To analyse the secretome of hUCB-MSCs co-cultured with primary neuronal cells impaired by Aβ42 peptide treatment, we used a human cytokine antibody array that can detect 507 different proteins. As a result, highly upregulated proteins based on signal intensity were identified (Supplementary Table S1). Among these, we focused on TSP-1, which is predominantly secreted by hUCB-MSCs, because TSP-1 was previously reported to promote the expression of synaptic proteins^23^.

**Figure 2:**
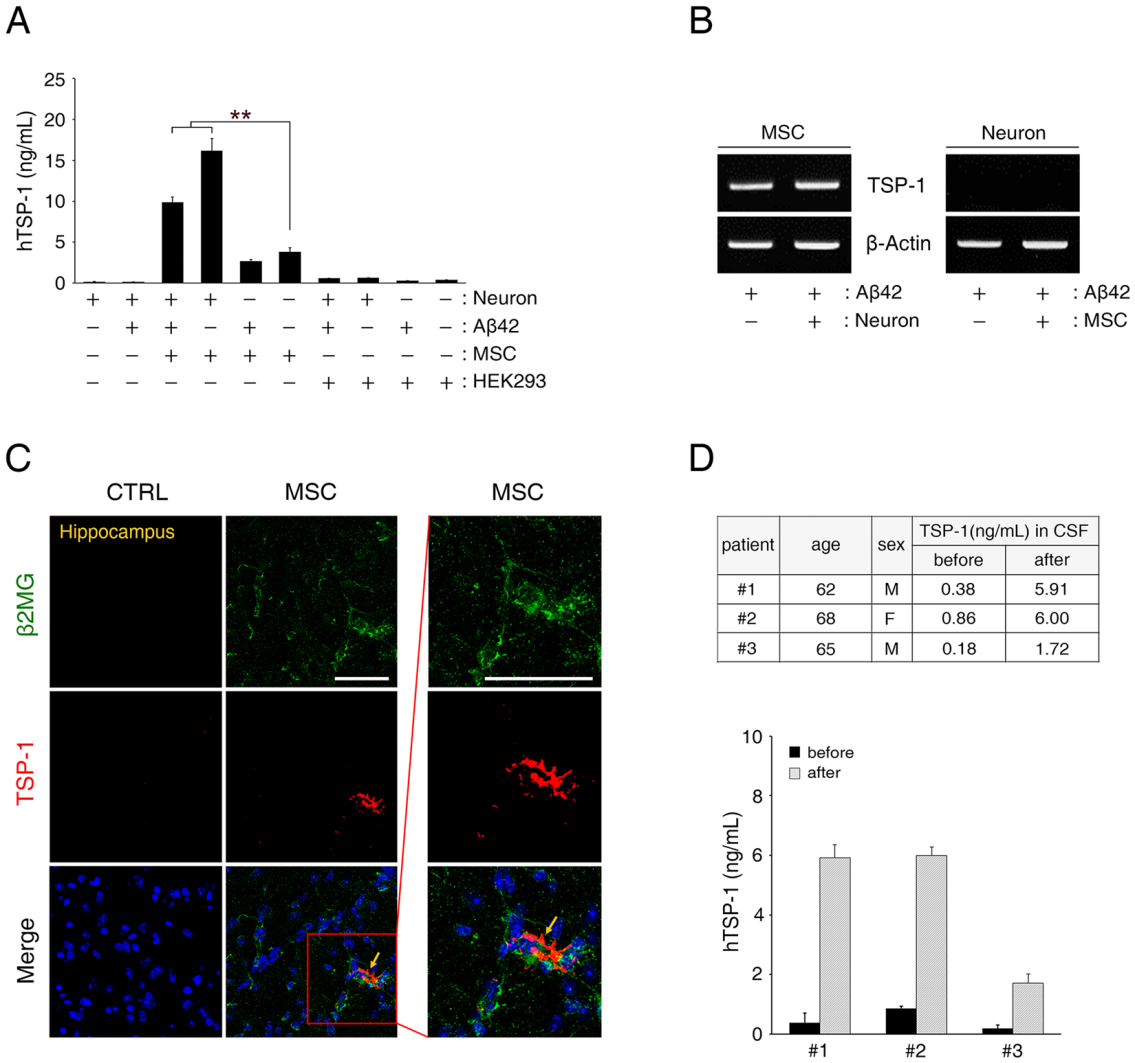
TSP-1 is a paracrine factor secreted by hUCB-MSCs under conditions of Aβ peptide-mediated AD pathogenesis. (**A**) Each conditioned medium used in the Transwell was analysed by an enzyme-linked immunosorbent assay (ELISA) to identify the relative quantity of secreted human TSP-1. hUCB-MSCs secreted TSP-1 in conditions with or without Aβ42 peptide treatment, unlike HEK293 cells (mean ± SEM, **p < 0.005, n = 3 per group). (**B**) To determine which cells secreted TSP-1 when co-cultured in the Transwell system, hippocampal neurons and hUCB-MSCs were co-cultured and then analysed by reverse transcriptase-polymerase chain reaction (RT-PCR) by using a TSP-1-specific primer. (**C**) Each tissue section was stained with DAPI, anti-β2MG and anti-TSP-1 antibodies. The confocal images show merged green (β2MG, human-specific) and red (TSP-1) co-localisation in the hippocampal region (Scale bars = 50 μm). The boxed area was magnified and merged to analyse the co-localisation of β2MG-labelled hUCB-MSCs and TSP-1-secreting cells. (**D**) Expression levels of TSP-1 in the cerebrospinal fluid of three AD patients were analysed by ELISA, before and one day after administration of hUCB-MSCs.

To verify the results of the cytokine antibody array, we performed an enzyme-linked immunosorbent assay (ELISA) to quantify the amount of human TSP-1 in the co-culture media. Even though the secretion of TSP-1 was slightly low because of the exposure to Aβ42 peptide treatment, the level of TSP-1 increased in the co-culture with hippocampal neurons as compared to TSP-1 level in the culture with only hUCB-MSCs (Fig. 2A).

To confirm the origin of TSP-1 secretion, after co-culturing primary hippocampal neurons with hUCB-MSCs under Aβ42 peptide treatment, both cell types were harvested separately, and the expression of TSP-1 mRNA was analysed by reverse transcriptase-polymerase chain reaction (RT-PCR). TSP-1 mRNA was found to be overexpressed in hUCB-MSCs, but not in hippocampal neurons, and expression of TSP-1 mRNA was increased in the co-culture of hUCB-MSCs with hippocampal neurons as compared to the culture with only hUCB-MSCs (Fig. 2B).

These results demonstrate that TSP-1 is secreted in hUCB-MSCs when they are co-cultured with primary hippocampal neurons under Aβ42 peptide treatment.

### Transplanted hUCB-MSCs secrete TSP-1 in an AD mouse model and elevate TSP-1 expression in the cerebrospinal fluid of AD patients

To further confirm the *in vivo* secretion of TSP-1 from hUCB-MSCs, we analysed the brains of 5XFAD mice via immunohistochemistry after the administration of hUCB-MSCs. The analysis showed that the transplanted hUCB-MSCs present in the brain parenchyma (green: β2-microglobulin-labelled hUCB-MSCs) co-localised with TSP-1 (red); and TSP-1-positive hUCB-MSCs were observed in the hippocampal region (Fig. 2C and Supplementary Fig. S1A).

Furthermore, to verify the effect of the hUCB-MSC transplantation, which modulated the expression of TSP-1 in three AD patients, we investigated the TSP-1 levels in the cerebrospinal fluid (CSF) of these patients using a human TSP-1-specific ELISA kit. For this analysis, we collected CSF pre- and post-administration of hUCB-MSCs from the three AD patients. We found that expression levels of TSP-1 significantly increased after transplantation of hUCB-MSCs into the patients (patient #1: 15.6-fold increase, #2: 6.98-fold increase, and #3: 9.56-fold increase) (Fig. 2D). Thus, these results suggest that hUCB-MSCs can secrete TSP-1 *in vivo* in both an AD mouse model and in AD patients.

### TSP-1 secreted from hUCB-MSCs rescue primary hippocampal neurons from Aβ peptide-induced loss in synaptic density

**Figure 3:**
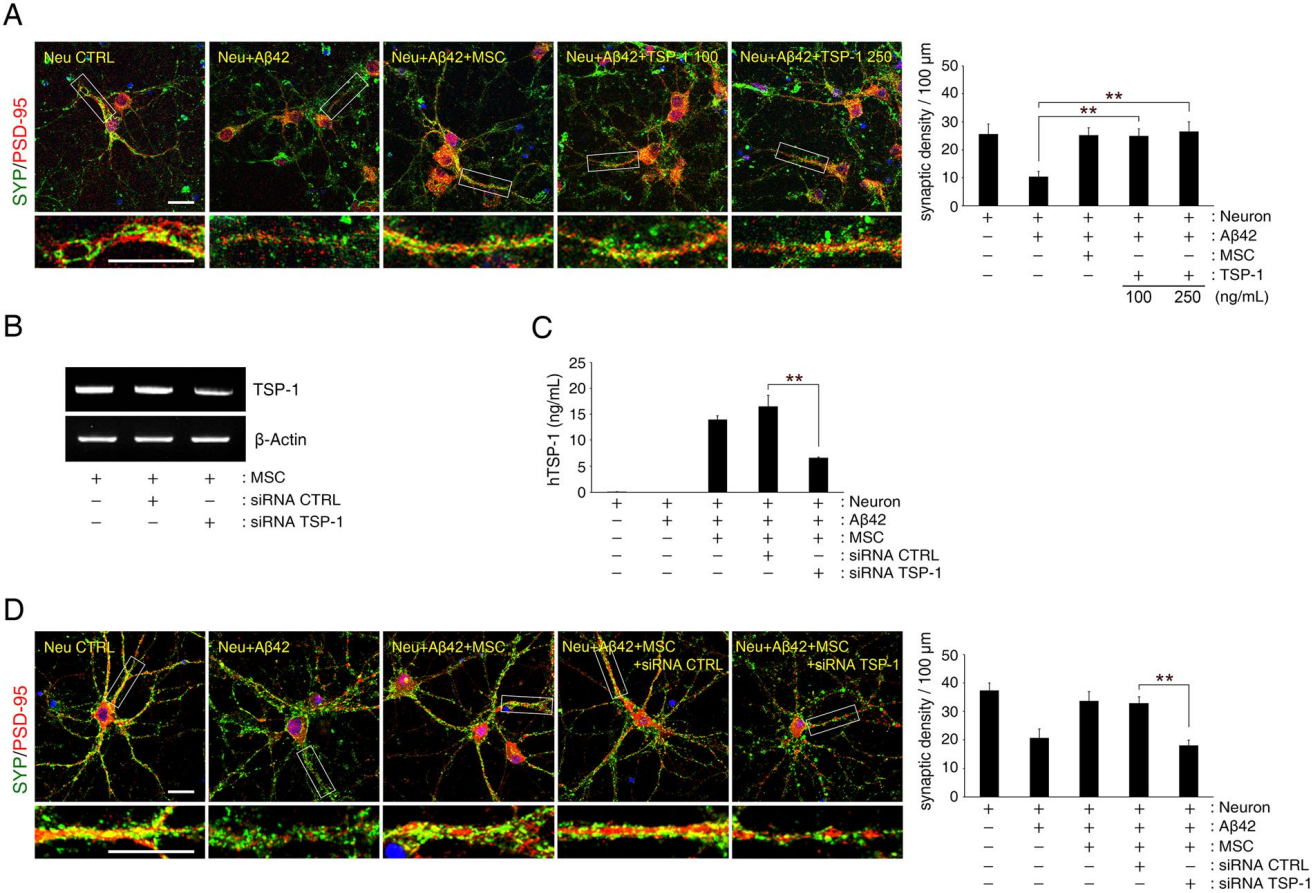
Aβ42 peptide-mediated synaptic dysfunction is mitigated by TSP-1 in hippocampal neurons. (**A**) Representative images of hippocampal neurons stained for pre-synaptic (SYP, green) and postsynaptic (PSD-95, red) proteins (Scale bar = 25 μm). Bottom insets (white boxes) show higher magnification. Quantification of synaptic density (number of synapses per 100 μm of dendritic length, n ≥ 30 dendrites) revealed that treatment with recombinant human TSP-1 protected hippocampal neurons from Aβ42 peptide-induced synaptic dysfunction (mean ± SEM, **p < 0.005 versus Aβ peptide-treated hippocampal neurons). (**B**,**C**) TSP-1-siRNA was transfected into hUCB-MSCs overnight, after which the cells were co-cultured with hippocampal neurons for 3 days. hUCB-MSCs were separately transfected with scrambled siRNA as a control. mRNA expression of TSP-1 was (**B**) analysed with RT-PCR, and (**C**) the relative quantity of secreted TSP-1 was determined using ELISA (mean ± SEM, **p < 0.005 versus control-siRNA-treated hUCB-MSCs). (**D**) Representative images of hippocampal neurons stained for pre-synaptic (SYP, green) and post-synaptic (PSD-95, red) proteins (Scale bar = 25 μm). Bottom insets (white boxes) show higher magnification. Quantification of synaptic density (number of synapses per 100 μm of dendritic length, n ≥ 30 dendrites) revealed that hUCB-MSCs with knockdown of TSP-1 by siRNA were not protected from Aβ42 peptide-induced synaptic dysfunction in hippocampal neurons. (mean ± SEM, **p < 0.005 versus control-siRNA-treated hUCB-MSCs).

Next, we attempted to determine the role of TSP-1 secreted from hUCB-MSCs in Aβ peptide-induced synaptic dysfunction. After adding either 100 or 250 ng/mL of human recombinant TSP-1 to Aβ42 peptide-treated primary hippocampal neurons, the loss in synaptic density was significantly reversed in these neurons (Fig. 3A). Moreover, TSP-1-specific siRNA (Fig. 3B,C), that knocked down TSP-1 expression in hUCB-MSCs, was used to validate this finding. It showed that, under this condition Aβ peptide-induced loss in synaptic density was not blocked in the primary hippocampal neurons (Fig. 3D). Thus, these results suggest that the role of TSP-1 secreted from hUCB-MSCs is to suppress Aβ peptide-induced synaptic-density loss in primary hippocampal neurons.

### TSP-1 secreted from hUCB-MSCs rescues primary hippocampal neurons from Aβ peptide-induced loss in synaptic density by mediating NLGN1 and α2δ-1 receptors

**Figure 4:**
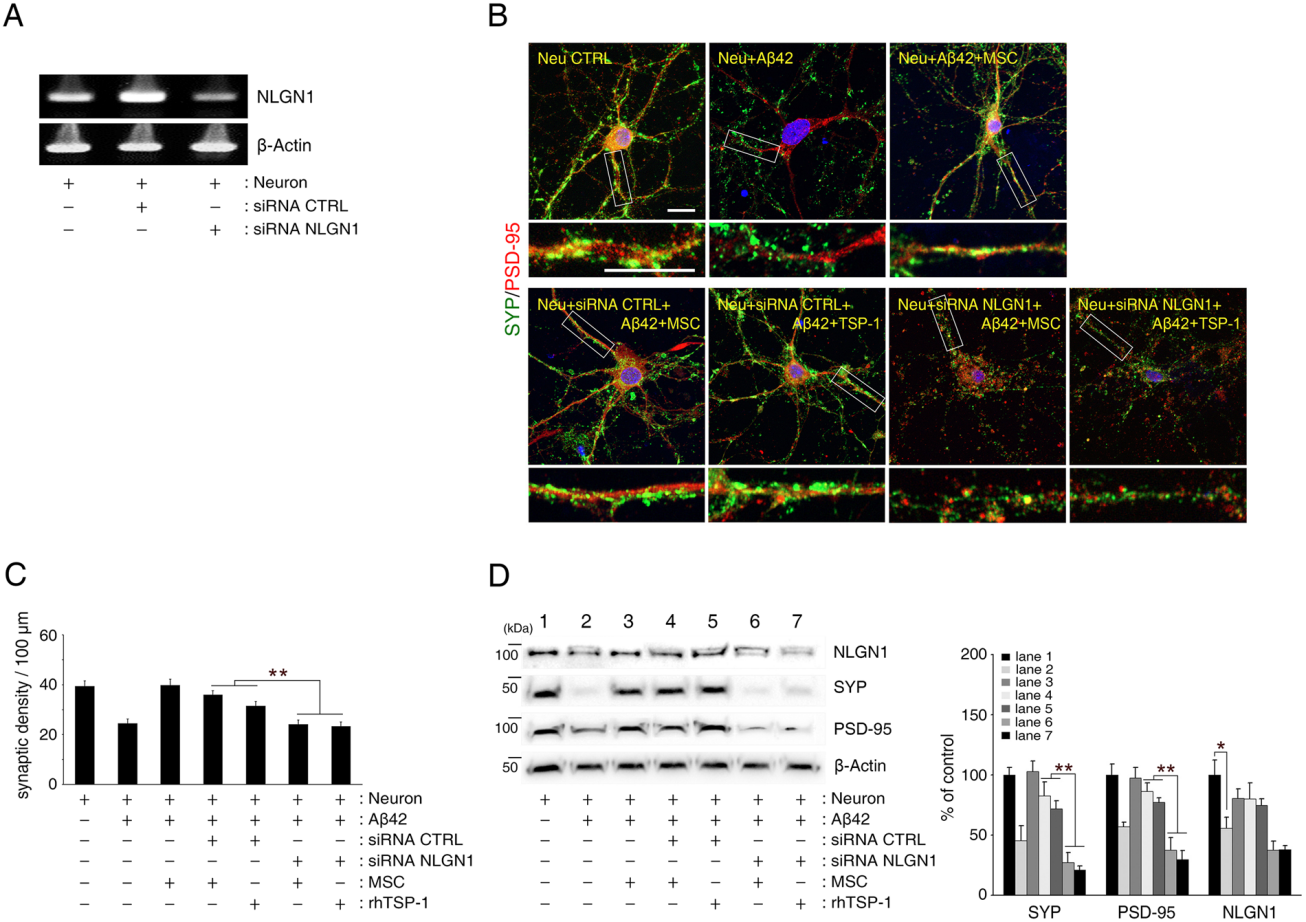
TSP-1 rescues hippocampal neurons from Aβ42 peptide-induced reduction of synaptic density by mediating the NLGN1 receptor. (**A**) NLGN1-siRNA was transfected into hippocampal neurons overnight, after which the cells were co-cultured with hUCB-MSCs or treated with recombinant human TSP-1 under Aβ42 peptide treatment. Hippocampal neurons were separately transfected with scrambled siRNA as a control. mRNA expression of NLGN1 was analysed using RT-PCR. (**B**) Representative images of hippocampal neurons stained for pre-synaptic (SYP, green) and post-synaptic (PSD-95, red) proteins (Scale bar = 25 μm). (**C**) Quantification of synaptic density (number of synapses per 100 μm of dendritic length, n ≥ 30 dendrites) revealed that suppression of NLGN1 by siRNA in the hippocampal neurons did not reverse Aβ42 peptide-induced synaptic dysfunction in the neurons, despite being co-cultured with TSP-1-secreting hUCB-MSCs or treatment with recombinant human TSP-1. (**p < 0.005 versus control-siRNA-treated hippocampal neurons). (**D**) In NLGN1-knockdown hippocampal neuronal cells, SYP and PSD-95 expression levels were determined by immunoblotting for the same conditions in (**B**, **C**). Immunoblotting also showed remarkably attenuated expression of SYP and PSD-95 in NLGN1-siRNA-treated hippocampal neurons and significantly decreased expression of NLGN1 in hippocampal neurons under Aβ42 peptide treatment. Right panel indicates densitometric quantification analysis. (mean ± SEM, **p < 0.005, *p < 0.05, n = 3 per group).

**Figure 5:**
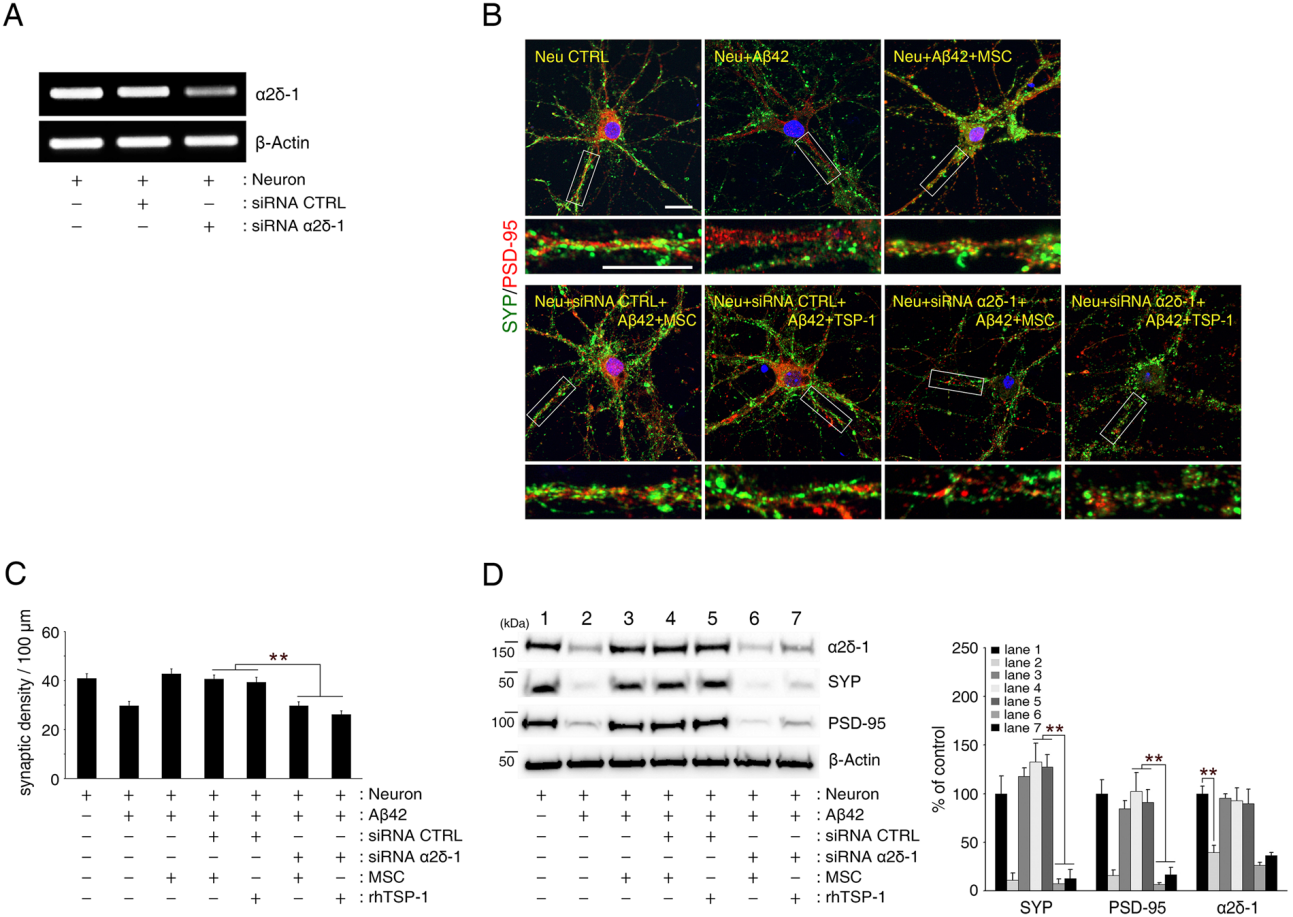
TSP-1 rescues hippocampal neurons from Aβ42 peptide-induced reduction in synaptic density by mediating the α2δ-1 receptor. (**A**) α2δ-1-siRNA was transfected into hippocampal neurons overnight, after which the cells were co-cultured with hUCB-MSCs or treated with recombinant human TSP-1 under Aβ42 peptide treatment. Hippocampal neurons were separately transfected with scrambled siRNA as a control. mRNA expression of α2δ-1 was analysed using RT-PCR. (**B**) Representative images of hippocampal neurons stained for pre-synaptic (SYP, green) and post-synaptic (PSD-95, red) proteins (Scale bar = 25 μm). (**C**) Quantification of synaptic density (number of synapses per 100 μm of dendritic length, n ≥ 30 dendrites) revealed that suppression of α2δ-1 by siRNA in the hippocampal neurons did not reverse Aβ42 peptide-induced synaptic dysfunction in hippocampal neurons, despite being co-cultured with the TSP-1 secreting hUCB-MSCs or treatment with recombinant human TSP-1. (**p < 0.005 versus control-siRNA-treated hippocampal neurons). (**D**) In α2δ-1-knockdown hippocampal neurons, SYP and PSD-95 expression levels were determined by immunoblotting for the same conditions in (**B**, **C**). Immunoblotting also showed remarkably attenuated expression of SYP and PSD-95 in α2δ-1-siRNA-treated hippocampal neurons and significantly decreased expression of α2δ-1 in hippocampal neurons under Aβ42 peptide treatment. Right panel indicates densitometric quantification analysis. (mean ± SEM, **p < 0.005, n = 3 per group).

To understand the underlying mechanism that prevents the loss of synaptic density based on the action of TSP-1 secreted from hUCB-MSCs, we investigated the involvement of known TSP-1 receptors such as neuroligin-1 (NLGN1) and α2δ-1^24^ in this process. We analysed the effect of NLGN1 and α2δ-1 knockdown (Figs 4A and 5A), on synaptic density in the co-culture of hUCB-MSCs and hippocampal neurons. The co-culture did not prevent the Aβ peptide-induced reduction in synaptic density (Figs 4B–D and 5B–D). Similarly, co-culture with knockdown of NLGN1 or α2δ-1 treated with TSP-1 (100 ng/mL) proteins also did not inhibit the Aβ peptide-induced reduction in synaptic density. Thus, these results indicated that TSP-1 secreted from hUCB-MSCs rescues hippocampal neurons from Aβ peptide-induced synaptic-density loss by mediating either NLGN1 or α2δ-1 receptors.

### TSP-1 secreted from hUCB-MSCs prevents Aβ peptide-induced reduction of NLGN1 and α2δ-1 expression

**Figure 6:**
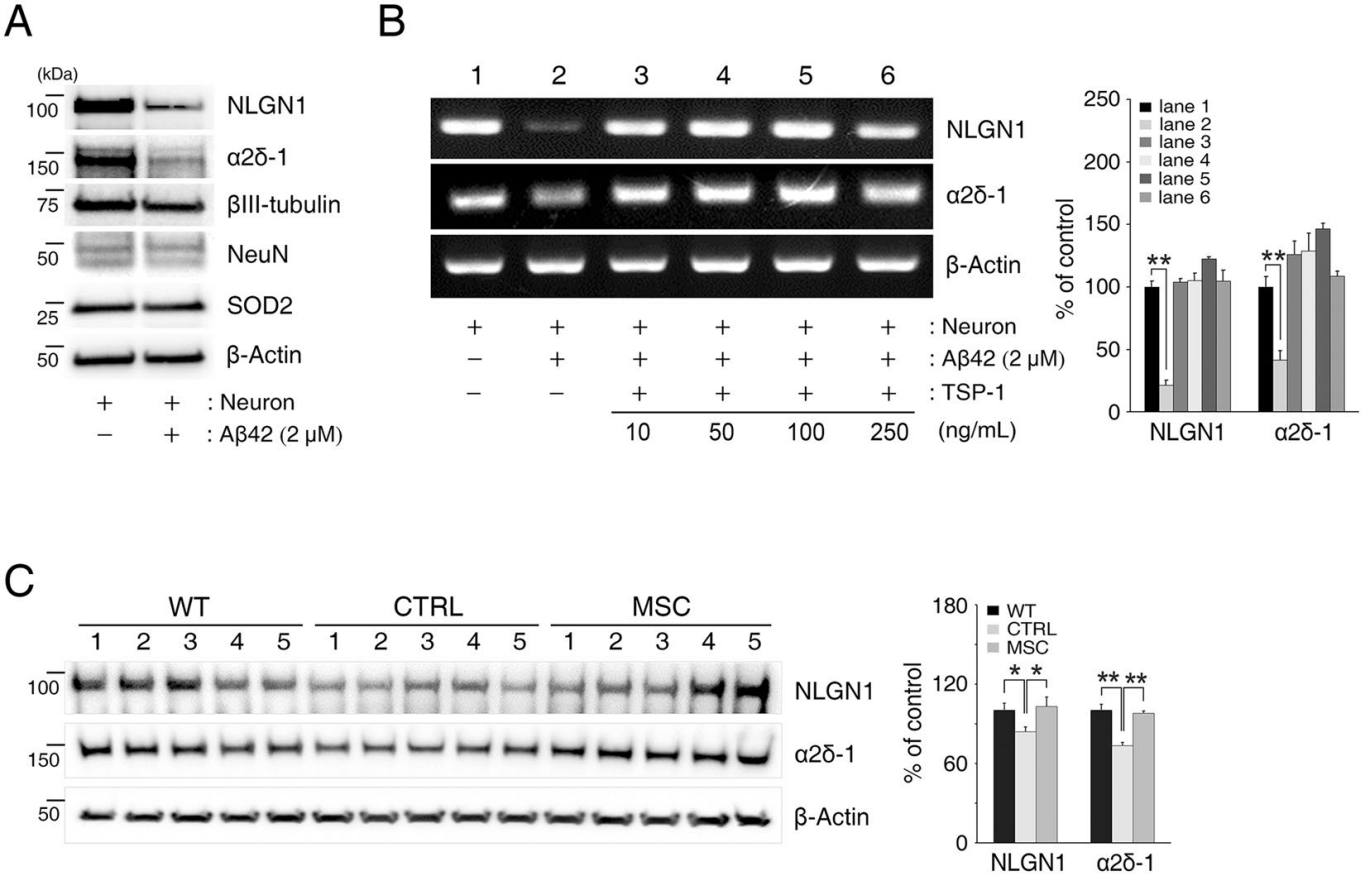
Aβ42 peptide-induced reduction in TSP-1 receptors was recovered by the addition of TSP-1 to hippocampal neurons. (**A**) After treatment of 2 μM Aβ42 peptide, hippocampal neurons were extracted and analysed by immunoblotting for NLGN1, α2δ-1, βIII-tubulin, NeuN, and SOD2 antibodies. β-Actin was used as a loading control. (**B**) Hippocampal neurons were treated with recombinant human TSP-1 (10, 50, 100, 250 ng/mL) under Aβ42 peptide treatment. mRNA expression levels of NLGN1 or α2δ-1 were analysed with RT-PCR. (mean ± SEM, **p < 0.005, n = 3 per group). (**C**) hUCB-MSC-administered mouse brains were extracted and analysed by immunoblotting with NLGN1 and α2δ-1 antibodies. β-Actin was used as a loading control. (WT: C57BL/6 J, CTRL: MEMα-administrated 5XFAD, MSC: hUCB-MSC-administered 5XFAD, n = 5 per group, *p < 0.05, **p < 0.005).

Interestingly, we observed that the expression of NLGN1 and α2δ-1 in hippocampal neurons was significantly decreased by the Aβ42 peptide treatment (NLGN1: 44.0% decrease, α2δ-1: 60.5% decrease) (Figs 4D and 5D, respectively). To further validate whether reduction of these receptors was associated with dedifferentiation or cell death of hippocampal neurons, we also checked the expression of mature neuron markers such as NeuN and βIII-tubulin, and a cell-death marker, superoxide dismutase-2 (SOD2), in the same *in vitro* system. Results showed that NLGN1 and α2δ-1 were similarly decreased; however, levels of NeuN, βIII-tubulin, and SOD2 remained unchanged (Fig. 6A). Likewise, mRNA expression of NLGN1 and α2δ-1 in hippocampal neurons was remarkably decreased by Aβ42 peptide treatment (NLGN1: 78.6% decrease, α2δ-1: 58.3% decrease) (Fig. 6B). This indicates that soluble Aβ42 peptide can induce a reduction in the expression of NLGN1 and α2δ-1, without causing cell death or other changes to hippocampal neurons.

Next, we found that the Aβ42 peptide-induced reduction in NLGN1 and α2δ-1 could be recovered by the addition of TSP-1 or in the co-culture with hUCB-MSCs (Figs 4D, 5D and 6B).

Based on the *in vitro* results, we further analysed the expression of NLGN1 and α2δ-1 in the brain of 6-month-old 5XFAD mice via immunoblotting. Age-matched C57BL/6 J mice were used as wildtype and compared to the results from 5XFAD mice. Results showed that the expression of these receptors in the 5XFAD mice was significantly decreased compared to that in the wildtype mice (NLGN1: 16.4% decrease, α2δ-1: 26.7% decrease). However, after administration of TSP-1 secreted by hUCB-MSCs to the 5XFAD mice, expression levels of these receptors recovered completely (Fig. 6C).

Collectively, these results show that soluble Aβ42 peptide specifically reduces the expression of NLGN1 and α2δ-1, which can be recovered by TSP-1 secreted from hUCB-MSCs.

## Discussion

AD is irreversible and intractable; therefore, the best treatment strategies for this disease include medical intervention at an early stage or a delay in disease progression^25^. Some recent studies have developed extensive diagnostic criteria or biomarkers for early-stage AD, such as mild cognitive impairment or prodromal AD^26^. Other studies have reported that soluble Aβ oligomer-induced synaptic dysfunction is an event that occurs in early-stage AD and is significantly correlated with an acceleration in cognitive impairment and memory loss^5,27^. Thus, resolving soluble Aβ peptide-induced synaptic dysfunction at an early stage of AD could be a strategically important target for therapy. The present study shows the new therapeutic potential for hUCB-MSCs in inhibiting Aβ peptide-induced synaptic dysfunction in *in vitro* and *in vivo* AD models.

We have already reported that the paracrine action of hUCB-MSCs results in an increase in synaptic activity of primary neuronal cells in a normal state. This phenomenon is regulated by GDF-15, which increases the release of synaptic vesicles and increases the stimulatory potential of synapses^20^.

In contrast, the present study aims to prove the hypothesis that hUCB-MSCs can recover from the loss in synaptic density induced by Aβ peptide in early-stage AD through their paracrine action. Synaptic dysfunction leads to memory impairment that is evident in early-stage AD, and persistent dysfunction may account for the neuronal loss typical in later stages of the disease^5^. We established an *in vitro* model for early-stage AD. Moreover, we selected a soluble Aβ peptide concentration that could affect synaptic function without inducing neuronal cell death, because soluble Aβ peptide can elicit synaptic impairment and neurotoxicity^28^. Furthermore, to evaluate only the paracrine effect of hUCB-MSCs, we excluded glial cells by treating the culture with AraC^29^ (Fig. 1A,B). As a result, we were able to confirm that soluble Aβ peptide-induced synaptic dysfunction could be suppressed by hUCB-MSCs through their paracrine action (Fig. 1C). In addition, the expression of synaptic markers such as SYP and PSD-95 increased after administration of hUCB-MSCs to 6-month-old 5XFAD AD mice^30^, which display cognitive dysfunction (Figs 2C and 1D).

Thus, these *in vitro* and *in vivo* results suggest that the paracrine action of hUCB-MSCs may play a therapeutic role in rescuing hippocampal neurons from synaptic dysfunction in an early-stage AD environment.

Next, the secretomes of hUCB-MSCs were analysed in an *in vitro* model established in this study via a protein array (Table S1), and TSP-1 was identified as a major paracrine factor suppressing soluble Aβ peptide-induced synaptic dysfunction.

TSP-1 is a matricellular glycoprotein that is a multifunctional protein affecting various biological functions^31^. In particular, TSP-1 is an important protein synthesised and secreted by astrocytes during the developmental phase of the CNS^32^. Moreover, it promotes synaptogenesis for neuronal function^33,34^ and increases synaptic density and activity^22^. TSP-1 is also involved in synaptic remodelling after injury^35^ and is a major contributor to astrocyte-regulated excitatory synapse formation^36^. TSP-1 secretion from astrocytes is reduced in the presence of Aβ peptide, which results in a loss of synaptic proteins^37^. Specifically, the mechanism of TSP-1 decrease in astrocytes has been reported to occur through autophagy activation^38^. Recent reports have found that TSP-1 expression was significantly reduced in the brains of AD patients and AD mouse models^39^; furthermore, TSP-1 deficiency induced a marked reduction in the frequency of excitatory synapses^36^. Nonetheless, few reports have been made regarding the inhibitory role of TSP-1 on synaptic dysfunction in an AD environment. Interestingly, the present study revealed that hUCB-MSCs persistently secreted TSP-1 and, remarkably, increased TSP-1 secretion levels in co-cultures with Aβ peptide-treated hippocampal neuronal cells (Fig. 2A,B). Similarly, TSP-1 secretion was observed in hUCB-MSCs transplanted into 5XFAD mice, and the expression of TSP-1 in the CSF of AD patients was significantly upregulated after the administration of hUCB-MSCs through the lateral ventricle (Fig. 2C,D).

Suppressing the expression of TSP-1 in hUCB-MSCs by TSP-1-specific siRNA did not reverse the Aβ peptide-induced decrease in synaptic density in the *in vitro* AD model established in this study; however, the decrease was reversed by the addition of exogenous TSP-1 (Fig. 3A–D). This implies that TSP-1 increases synaptic density, not only in a normal environment, but also in an AD environment. The treatment of TSP-1 alone was able to reverse the damage to synaptic density caused by the presence of Aβ peptide, an effect that was similar with treatment of TSP-1-secreting hUCB-MSCs. The co-culture of TSP-1-knocked down hUCB-MSCs and Aβ peptide treated hippocampal neurons showed similar damage to synaptic density as was seen in the culture of hippocampal neurons treated with Aβ peptide alone. These findings indicate that TSP-1 is a dominant factor in hUCB-MSCs associated with the rescue effect on Aβ peptide-mediated loss of synaptic density. Thus, our findings suggest that TSP-1 secreted by hUCB-MSCs, is a major paracrine factor mediating recovery from Aβ peptide-induced synaptic dysfunction in hippocampal neurons.

Together, the expression of TSP-1 by astrocytes, which is reduced by the presence of Aβ peptide in AD patients, can be augmented by transplanting hUCB-MSCs, which exhibit a therapeutic effect by rescuing hippocampal neurons from Aβ peptide-induced synaptic dysfunction.

NLGN1 and α2δ-1 are well-known receptors for TSP-1 involved in synapse formation^36^. NLGN1 is a post-synaptic protein that mediates the synaptogenic effect of TSP-1 in hippocampal neurons^33^. α2δ-1 is a subunit of the L-type calcium channel complex that is ubiquitously expressed and highly expressed in CNS neurons. The over-expression of α2δ-1 has been reported to increase synaptogenesis *in vitro* and *in vivo* and is required for TSP-induced synapse formation *in vitro*^40^. α2δ-1 is primarily located in pre-synaptic neurons, and the binding of TSP-1 to the α2δ-1 receptor promotes the assembly of excitatory synapses^24^.

We aimed to determine whether the two receptors were involved in the recovery mechanism of TSP-1 secreted from hUCB-MSCs to reverse soluble Aβ peptide-induced synaptic dysfunction. When suppressing the expression of these two receptors by α2δ-1- or NLGN1-specific siRNA treatment in hippocampal neurons, the exogenous TSP-1 treatment and co-culture with TSP-1-secreted hUCB-MSCs did not show a rescue effect on the hippocampal neurons with Aβ peptide-induced loss in synaptic density (Figs 4 and 5). Following these results, we concluded that TSP-1 secreted from hUCB-MSCs can mitigate Aβ peptide-induced synaptic dysfunction, not only through the α2δ-1 receptor^38^, as was previously known, but also through the NLGN1 receptor.

The reduction of major synaptic receptors in neurons caused by the presence of Aβ peptide has been documented in previous studies. For example, glutamate receptors, including types of NMDA (N-methyl-D-aspartate) and AMPA (2-amino-3- (3-hydroxy-5-methyl-isoxazol-4-yl) propanoic acid) receptors, primarily participate in synaptic targeting via soluble Aβ peptide. Binding of soluble Aβ peptide dysregulates the activity and decreases the surface expression of NMDA and AMPA glutamate receptors, which results in damaging the signalling pathway associated with synaptic plasticity^41^.

Although no report has described the change in expression of the TSP-1 receptor because of Aβ peptide in hippocampal neurons, we found a decrease in the expression of α2δ-1 and NLGN1 caused by the presence of soluble Aβ peptide in an *in vitro* model reflecting Aβ peptide-induced synaptic dysfunction. However, exogenous treatment with TSP-1 and co-culture with TSP-1-secreting hUCB-MSCs mitigated the Aβ peptide-induced loss in synaptic density and reversed the Aβ peptide-induced downregulation of α2δ-1 and NLGN1 receptors in the hippocampal neurons (Figs 4D, 5D and Fig. 6B). Moreover, expression levels of α2δ-1 and NLGN1 receptors, which are low in the 5XFAD AD mouse model, increased after the administration of hUCB-MSCs that secrete TSP-1 (Fig. 6C). Although details of these mechanisms should be investigated further, there are two possible explanations for these findings. Firstly, the Aβ clearance effect of TSP-1 could lead to maintaining the expression of NLGN1 and α2δ-1 receptors in the presence of soluble Aβ peptide. This possibility is supported by the result that administration of TSP-1 reduced the level of Aβ in the 5XFAD AD mouse model (Supplementary Fig. S2). In fact, transplanted TSP-1-secreting hUCB-MSCs have been shown to migrate to regions of Aβ deposition^19^ (Supplementary Fig. S1B). Thus, the reduction of Aβ by TSP-1 could alleviate the downregulation of NLGN1 and α2δ-1 receptors. Secondly, TSP-1 signalling could induce the expression of NLGN1 or α2δ-1 receptors in hippocampal neurons with soluble Aβ peptide. Indeed, TSP-1 has been shown to regulate the Nrf2 (nuclear factor erythroid-derived 2-related factor 2) pathway^42^. NLGN1 has been reported to be one of the downstream target genes of Nrf2 in response to oxidative stress^43^. Thus, TSP-1 can induce the expression of these receptors via the Nrf2 pathway depending on oxidative stress in the presence of soluble Aβ peptide.

The knockdown of NLGN1 and α2δ-1 receptors resulted in significant loss of synaptic density even without soluble Aβ peptide (Supplementary Fig. S3). These findings indicate that NLGN1 and α2δ-1 are necessary to maintain synaptic activity and function^40,44^. Furthermore, co-cultures with TSP-1 knockdown hUCB-MSCs decreased the expression of these receptors in hippocampal neurons with soluble Aβ peptide (Supplementary Fig. S4). These results imply that the reduction of TSP-1 signalling can lead to decreased expression of NLGN1 and α2δ-1 receptors. Collectively, these results and the proposed hypotheses suggest that secreted TSP-1 by hUCB-MSCs transplanted into an AD model regulate the expression of NLGN1 and α2δ-1 receptors, in turn, leading to maintaining synaptic activity and function of hippocampal neurons.

Finally, our results suggest that TSP-1 from hUCB-MSCs can reverse the Aβ peptide-induced downregulation of α2δ-1 and NLGN1 receptors and, in turn, mediate the recovery from Aβ peptide-induced synaptic dysfunction in hippocampal neurons.

We confirmed that TSP-1 from hUCB-MSCs can rescue hippocampal neurons from Aβ peptide-induced synaptic dysfunction by analysing the change in expression of synaptic density markers, such as SYP and PSD-95. However, further studies using the same model could reveal synaptic transmission in response to the action potential of hippocampal neurons. This study type could reveal the functional aspects of and recovery from cognitive impairment in the AD mouse model by using behavioural testing.

**Figure 7:**
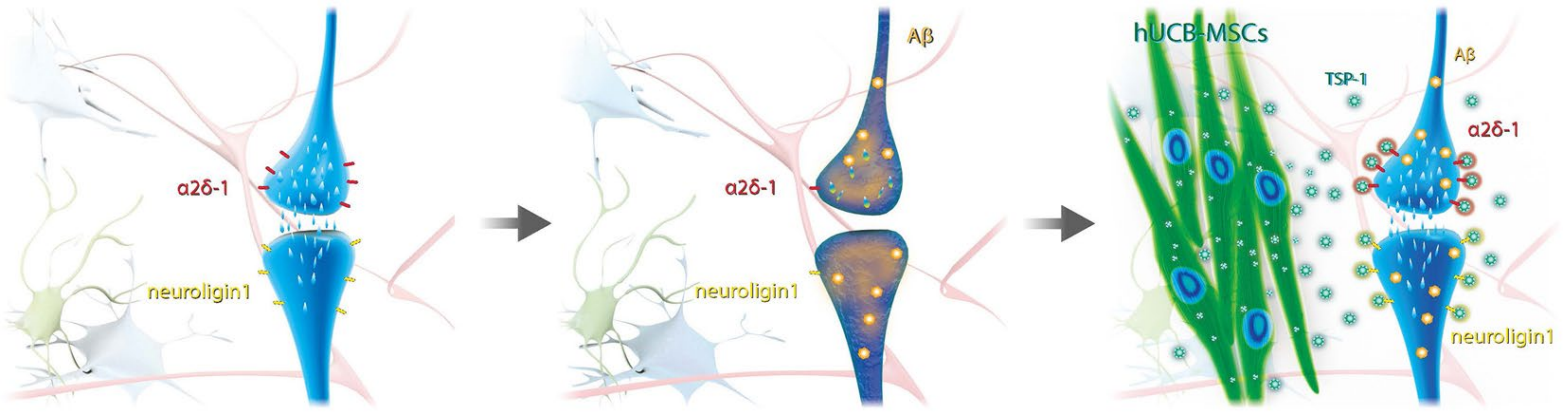
Schematic diagram of therapeutic effect of hUCB-MSCs on Aβ peptide-induced synaptic dysfunction. Oligomeric low-concentration of Aβ peptide induced loss in synaptic function and reduced expression of TSP-1 receptors in hippocampal neurons. Interestingly, hUCB-MSC-derived TSP-1 promoted elevation of synaptic function via TSP-1 receptors as well as recovery of TSP-1 receptors such as NLGN1 and α2δ-1. Consequently, TSP-1-secreting hUCB-MSCs may reveal therapeutic effects on Aβ peptide-induced synaptic dysfunction.

In conclusion, we discovered that the paracrine action of hUCB-MSCs could mitigate Aβ peptide-induced synaptic dysfunction, which is closely related to cognitive and memory impairment. In particular, we found that the TSP-1 protein, whose secretion is increased in hUCB-MSCs under AD conditions, is a major paracrine factor for maintaining the synaptic density of neuronal cells through the α2δ -1 and NLGN1 receptors (Fig. 7). Additionally, we found that TSP-1 secreted by hUCB-MSCs can recover the expression levels of α2δ-1 and NLGN1 receptors, coupling partners in an AD environment. These results suggest that TSP-1 secretion by hUCB-MSCs could have a therapeutic effect on cognitive impairment and memory loss in early-stage AD, thus providing a new and exciting strategic option for AD treatment.

## Methods

### Ethical Statement

The animal study was approved by the Institutional Review Board of MEDIPOST Co., Ltd. All the animal procedures were conducted in accordance with institutional guidelines and approved protocols. The animal study was reviewed and approved by the Institutional Animal Care and Use Committee of the Samsung Biomedical Research Institute (SBRI). The SBRI is an Association for Assessment and Accreditation of Laboratory Animal Care International (AAALAC International)-accredited facility, and abides by the Institute of Laboratory Animal Resources (ILAR) guidelines.

In accordance with the guidelines approved by the Institutional Review Board of MEDIPOST Co., Ltd., neonatal human umbilical cord blood (hUCB) was collected from umbilical veins with informed maternal consent.

For all patients, informed consent in the CSF study was obtained according to sampling protocols that were approved by the Institutional Review Board of Samsung Medical Center (SMC), Korea. All the procedures were conducted in strict compliance with institutional guidelines and approved protocols.

### Preparation of hUCB-MSC culture

The isolation and culturing procedures of hUCB-MSCs have been described in previous report^45^. Mononuclear cells were isolated from the hUCB by centrifugation on a Ficoll-Hypaque gradient (density: 1.077 g/cm^3^; Sigma, St. Louis, MO, USA). Separated mononuclear cells were washed, suspended in minimum essential medium alpha (MEMα) supplemented with 10% (v/v) foetal bovine serum and 50 mg/mL gentamicin (Gibco, Carlsbad, CA, USA), and seeded at a concentration of 5000 cells per centimetre squared in culture flasks. The cultures were maintained at 37 °C in a humidified 5% CO_2_ atmosphere with twice-weekly medium changes. After 5 days, when the monolayer of fibroblast-like adherent cell colonies had reached 80% confluence, the cells were detached with 0.25% Trypsin- ethylenediaminetetraacetic acid (1X) (Gibco), washed with phosphate-buffered saline (PBS), re-suspended in culture medium and sub-cultured. In all experiments, hUCB-MSCs used were at passage 6.

### Primary culture, protein treatment and MSC co-culture system

Pregnant ICR mice were purchased from SAMTACO (SAMTACO Bio Korea, Osan, Korea). Hippocampal tissue was dissected from the brain of embryonic-day-18 mice, and cells were mechanically dissociated in Ca^2+^/Mg^2+^ free Hank’s balanced salt solution. The cells were seeded at a density of 2.5 × 10^4^/cm^2^ on coverslips coated with 0.2 mg/mL of poly-L-lysine (Sigma). The neurons were allowed to proliferate in the presence of 8 ng/mL basic fibroblast growth factor (bFGF; Sigma) in a neurobasal media supplemented with 2% B-27, L-glutamine and penicillin-streptomycin (Gibco). After 3 days (DIV 3), media change was performed with cytosine-arabinoside (AraC; Sigma) at a concentration of 0.65 μM to stop glial cells from growing without bFGF^29^. On DIV 7, after a medium change, primary hippocampal neurons were treated with 100–250 ng/mL of human recombinant TSP-1 (R&D Systems, Minneapolis, MN, USA) or were co-cultured with hUCB-MSCs of 2.0 × 10^4^/cm^2^ density for 3 days using Transwell system (Corning Inc., Corning, NY, USA). Human Aβ42 peptide was purchased from AnaSpec (AnaSpec Inc., Fremont, CA, USA) and dissolved according to manufacturer’s instructions to ensure solubility. Lyophilised Aβ42 peptide powder was re-suspended in 1.0% NH_4_OH and then immediately diluted with PBS to a working concentration of approximately 100 µM. To induce synaptic dysfunction, Aβ42 peptide treatment was performed as described previously^6^.

### Treatment with siRNA

TSP-1 siRNA and scrambled control siRNA were purchased from Thermo Fisher Scientific Inc. (Waltham, MA, USA). The TSP-1-siRNA (25 nmol/L) was treated with Lipofectamine^TM^ 3000 (Thermo Fisher Scientific Inc.) in the hUCB-MSC culture 1 day before being co-cultured with hippocampal neurons. NLGN1 and α2δ-1 siRNA were purchased from Dharmacon (Lafayette, CO, USA). In hippocampal neurons (DIV 6), NLGN1-siRNA (25 nmol/L) or α2δ-1-siRNA (25 nmol/L) were treated with DhamaFECT (Dharmacon) under serum-free media. The hippocampal neurons were cultured in complete media overnight before treatment with TSP-1 protein or co-cultured with hUCB-MSCs. Each treatment with siRNA was performed following the manufacturer’s protocols. Control siRNA with a minimum of 4 mismatches to any human, mouse, or rat gene were used.

### Cytokine antibody array

Growth medium was collected from the primary neurons and co-cultures of neurons and hUCB-MSCs with or without Aβ42 peptide treatment. In order to detect the proteins secreted from the hUCB-MSCs, a biotin-label-based Human Cytokine Antibody Array (RayBiotech. Inc., Norcross, GA, USA) was conducted according to the manufacturer’s protocol. Each conditioned medium was mixed with a biotin-labelling reagent. The glass chips were incubated in blocking buffer at room temperature and in each biotin-labelled conditioned medium for 2 h at room temperature (RT). After washing, the glass chips were incubated with streptavidin-conjugated fluorescent dye for 2 h at RT. After another wash and final drying step, signals from the glass chips were detected with a VIDAR Revolution 4200 Laser Scanner (VIDAR Systems Corp., Herndon, VA, USA), and normalised signal intensity was acquired with Analysis Tool software (RAYBIO® ANALYSIS TOOL) provided by RayBiotech.

### Immunoblot analysis, immunocytochemistry, and immunohistochemistry

For standard immunoblotting analysis, primary hippocampal neurons were lysed in buffer containing 9.8 M urea, 4% CHAPS, 130 mM dithiothreitol, 40 mM Tris-Cl and 0.1% sodium dodecyl sulfate (SDS) and prepared by ultrasonication (Branson Ultrasonics, Danbury, CT, USA). Amount of protein was measured by the Bradford assay (Bio-Rad Laboratories, Inc., Hercules, CA, USA). Cell lysates were run on SDS-polyacrylamide gel electrophoresis and transferred to nitrocellulose membranes or polyvinylidene difluoride. The membranes were blocked in 5% skim-milk in Tris-buffered saline Tween-20 **(**TBST) for 1 h and incubated in primary antibodies for indicated proteins in 5% skim-milk in TBST at 4 °C. The membranes were washed three times with TBST and incubated with horseradish peroxidase-conjugated anti-mouse or anti-rabbit secondary antibodies in 5% skim-milk at room temperature for 2 h. ECL detection kit (GE Healthcare Life Sciences, Little Chalfont, UK) was used for visualisation. Anti-mouse SYP, anti-rabbit PSD-95, anti-rabbit MAP2, anti-mouse NeuN, anti-rabbit beta III tubulin, anti-rabbit SOD2, anti-mouse α2δ-1, anti-mouse TSP-1 and anti-mouse NLGN1 were purchased from Abcam (Cambridge, UK). Anti-mouse β-actin was purchased from Sigma-Aldrich Co. Anti-mouse Aβ42 (MOAB-2) was purchased from Novus Biologicals (Littleton, CO, USA). Anti-rabbit beta-2 microglobulin was purchased from Abnova (Taipei City, Taiwan).

For immunocytochemistry, hippocampal neuronal cells were fixed for 10 min in 4% PFA for 3 days after the co-culture. After 3 washes with Dulbecco’s phosphate-buffered saline (DPBS) (Corning Inc., Corning, NY, USA), permeabilization was performed for 10 min with 0.3% Tween 20. After blocking for 1 h in a 5% normal goat serum and 5% normal horse serum (VECTOR Laboratories, Burlingame, CA, USA) solution, the cells were stained with SYP and PSD-95 (Abcam) antibodies overnight at 4 °C. Biotinylated anti-mouse (VECTOR Laboratories) and Cy3-anti-rabbit (Jackson ImmunoResearch Laboratories, West Grove, PA, USA) were added for 2 h, DTAF-conjugated streptavidin (Jackson ImmunoResearch Laboratories) was consequently added after a PBS wash. Images of neurons stained with SYP and PSD-95 were obtained using a LSM 700 confocal microscope (ZEISS, Jena, Germany). Since only co-localisation of pre-synaptic and post-synaptic markers represent active synapses, we analysed synaptic puncta containing SYP and PSD-95.

### Synaptic density (synaptic puncta) analysis

A co-localisation program, SynapCountJ as an ImageJ plugin (http://imagejdocu.tudor.lu/doku.php?id=plugin:utilities:synapsescountj:start), was used to assess synaptic density by evaluating the co-localisation of pre- and post-synaptic markers as previously described^46^. SynapCountJ semi-automatically quantified the number of synapses in the neuron cultures, and synaptic density was calculated as density of synapses per 100 microns. For analysis, more than 30 dendrites were used two or more times in independent cultures. Dendrites and regions of interest were randomly chosen and the threshold of intensity was fixed during the analysis.

### Animal studies

For the animal studies, 5XFAD mice (B6SJL-Tg(APPSwFlLon,PSEN1*M146L*L286V)6799Vas/Mmjax); The Jackson Laboratory, Bar Harbor, ME, USA) were purchased from the Jackson labs and maintained by the SBRI until they were 6-month-old. 5XFAD mice develop the amyloid pathology at 2 months-of-age and cognitive impairment at 4 to 6 months-of-age^47^. Mice were cannulated, and hUCB-MSCs were transplanted via an intracerebroventricular (ICV) method with reference to the previous study^48^. 5XFAD mice were anaesthetised with intraperitoneal injections of Zoletil™ (VIRBAC Corp., Fort Worth, TX, USA) or Rompun™ (xylazine) (BAYER KOREA, Seoul, Korea). They were then fixed in a stereotaxic apparatus (Stoelting Co., Wood Dale, IL, USA) for hUCB-MSC transplantation; 15 μL of hUCB-MSCs (1 × 10^5^ cells) was administrated via a cannula into the lateral ventricle (AP: −0.22, ML: 1.0, DV: −2.1 mm, with reference to the bregma) of the mice in the stereotaxic apparatus with a sterile Hamilton syringe fitted with a 26-gauge needle (Hamilton Company, Reno, NV, USA). The cell suspension was delivered at 1.0 μL/min using a Pump 11 Elite micro infusion syringe pump (Harvard Apparatus., Holliston, MA, USA).

### Human study and human CSF

Human study in the Phase-I/IIa clinical trial (ClinicalTrial.gov Identifier: NCT02054208) aims to evaluate the safety and efficacy of administration of hUCB-MSCs in patients with AD. hUCB-MSCs were administered three times into the lateral ventricle via an Ommaya reservoir at 4 week intervals in patients with AD.

Human CSF of three patients participating in the Phase-I/IIa clinical trial was obtained via an Ommaya reservoir for analysis according to clinical protocol. Patient information is presented in Fig. 2D.

### RT-PCR

Total RNA from cell lysates were isolated using Trizol reagent (Life Technologies, Carlsbad, CA, USA) according to the protocol recommended by the manufacturer. cDNA was synthesised from the total RNA with the SuperScript® III Reverse transcriptase kit and oligo (dT) primers (Life Technologies). For each reaction, 1 µL of 1 µg cDNA and 0.1 µL of 10 pmol primer set were combined with 2X master mix for a final volume of 20 µL. The reactions were analysed using a ProFlex PCR System (Thermo Fisher Scientific) at the following settings: 120 s incubation at 95 °C, followed by a three-step cycling program with 30 cycles of 30 s at 95 °C, 30 s at 55 °C, 60 s at 72 °C, and an additional cycle of 5 min at 72 °C.

PCR reactions were performed with the following primers.

$$\begin{array}{ll}{\rm{TSP}}-1 & {\rm{F}}:{\rm{CAT}}\,{\rm{CTT}}\,{\rm{TGA}}\,{\rm{ACT}}\,{\rm{CAC}}\,{\rm{CGG}}\,{\rm{GGC}}\\  & {\rm{R}}:{\rm{GTG}}\,{\rm{AAG}}\,{\rm{ACG}}\,{\rm{CTT}}\,{\rm{TGG}}\,{\rm{ATG}}\,{\rm{GGG}}\\ {\rm{alpha}}\,2\,{\rm{delta}}\,{\rm{sub}}-1 & {\rm{F}}:{\rm{AGA}}\,{\rm{GTG}}\,{\rm{AGC}}\,{\rm{CAG}}\,{\rm{GCA}}\,{\rm{GCC}}\,{\rm{AA}}\\  & {\rm{R}}:{\rm{GCC}}\,{\rm{AAA}}\,{\rm{CAC}}\,{\rm{TTG}}\,{\rm{CCA}}\,{\rm{CAG}}\,{\rm{CA}}\\ {\rm{Neuroligin}}\,1 & {\rm{F}}:{\rm{CAT}}\,{\rm{CTT}}\,{\rm{GGC}}\,{\rm{TTT}}\,{\rm{TGC}}\,{\rm{AGC}}\,{\rm{CC}}\\  & {\rm{R}}:{\rm{CGG}}\,{\rm{TCC}}\,{\rm{GAA}}\,{\rm{GAA}}\,{\rm{CCA}}\,{\rm{CCT}}\,{\rm{CA}}\\ {\rm{\beta }}-{\rm{actin}} & {\rm{F}}:{\rm{GAC}}\,{\rm{CTT}}\,{\rm{CAA}}\,{\rm{CAC}}\,{\rm{CCC}}\,{\rm{AGC}}\,{\rm{CA}}\\  & {\rm{R}}:{\rm{TAG}}\,{\rm{CTC}}\,{\rm{TTC}}\,{\rm{TCC}}\,{\rm{AGG}}\,{\rm{GAG}}\,{\rm{GA}}\end{array}$$

### ELISA

An ELISA was performed according to the manufacturer’s instructions. For measurement of secreted TSP-1 in conditioned media, a TSP-1 Duoset ELISA development system (R&D Systems) was used. Results were analysed at 450 nm by using a VERSAmax microplate reader (Molecular Devices, Sunnyvale, CA, USA).

### TUNEL staining (assay)

Primary hippocampal neurons were fixed for 1 h in a 4% paraformaldehyde and PBS solution at room temperature, and then permeabilised with 0.1% Triton X-100 in a 0.1% sodium citrate solution for 2 min on ice. The TUNEL assay (Roche Applied Science, Penzberg, Germany) was carried out following the manufacturer’s instructions.

### Statistics

All data are represented as means ± SEM and were analysed by using Student’s t-tests when appropriate. Significant levels were set at *p < 0.05, **p < 0.005.

### Data Availability

The datasets generated during the current study are available from the corresponding author on reasonable request.

## Electronic supplementary material

Supplementary Information
