# Supplementary material for: Thrombospondin-1 secreted by human umbilical cord blood-derived mesenchymal stem cells rescues neurons from synaptic dysfunction in Alzheimer’s disease model
Source: Sci Rep. 2018 Jan 10;8:354. doi: 10.1038/s41598-017-18542-0 (PMC5762817; doi:10.1038/s41598-017-18542-0)
Supplement: Supplementary file 1 — Supplementary Information [file 41598_2017_18542_MOESM1_ESM.pdf]

Supplementary Information

Thrombospondin-1 secreted by human umbilical cord blood-derived mesenchymal stem cells rescues neurons from synaptic dysfunction in Alzheimer's disease model

Running head: TSP-1 from hUCB-MSCs as a major enhancer for synaptic density

Dong Hyun Kim[1,2 §], Hoon Lim[1§], Dahm Lee[1], Soo Jin Choi[1], Wonil Oh[1], Yoon Sun Yang[1], Jeong Su Oh[2], Hyun Ho Hwang[3] and Hong Bae Jeon[1*]

[1]*Biomedical Research Institute, R&D Center, MEDIPOST Co., Ltd, Gyeonggi-do, Republic of Korea*

[2]*Department of Genetic Engineering, College of Biotechnology and Bioengineering, Sungkyunkwan University, Suwon, Republic of Korea*

[3]*King Abdullah University of Science and Technology, Thuwal, Saudi Arabia*

[§]These authors contributed equally to the study.

***Corresponding author:** Hong Bae Jeon, Ph.D. Biomedical Research Institute, MEDIPOST Co., Ltd, Gyeonggi-do 463-400, Republic of Korea; E-mail: jhb@medi-post.co.kr; Phone: +82-2-3465-6772; Fax: +82-2-475-1991.

RayBiotech® Human Cytokine Antibody Arrays -- Biotin Label Based Antibody Array I G Series

Supplementary Table 1. Nineteen proteins upregulated in supernatant from the hUCB-MSCs co-cultured with primary neuronal cells under Aβ42 treatment.

Protein Name	1 Neuron CTRL	2 Neuron+Aβ42	3 Neuron+Aβ42+MSC	4 Neuron+MSC	3-4-2-1
	Signal[a]	Signal[a]	Signal[a]	Signal[a]	Substantial signal[b]
Activin A	421.33	545.72	31,989.09	25,631.24	5390.80
Decorin	249.00	311.35	1,494.78	765.45	168.97
Dkk-1	262.00	309.82	1,733.12	1,110.81	50.48
Dkk-3	224.67	305.63	1,453.58	798.18	125.10
Follistatin	385.67	461.88	2,445.19	1,446.89	150.76
Galectin-3	343.67	376.13	2,502.08	1,718.97	63.31
GDF-15	424.33	529.33	3,824.72	2,383.31	487.75
Glypican 3	349.67	421.10	8,166.83	7,306.25	89.81
ICAM-5	369.67	436.34	2,673.73	1,740.47	127.25
IGFBP-rp1 / IGFBP-7	399.00	488.55	33,922.79	32,439.24	596.00
MFRP	256.33	312.11	1,880.73	1,193.37	118.92
PDGF-AA	400.67	455.78	11,683.58	10,270.37	556.76
PF4 / CXCL4	217.67	307.16	1,913.10	1,334.54	53.74
Progranulin	245.33	321.26	4,031.68	3,146.32	318.76
SPARC	299.00	367.37	7,326.27	5,598.03	1061.87
Thrombospondin (TSP)	516.67	545.72	4,245.99	1,764.89	1418.71
Thrombospondin-1	318.00	370.04	3,488.30	2,199.15	601.11
WISP-1 / CCN4	320.67	350.60	2,054.83	1,214.37	169.19
XEDAR	246.00	301.82	1,948.41	1,247.10	153.49

[a] The presented values signified the normalised signal intensity of each protein expressed in each culture medium condition.

Normalised signal intensity data were acquired using the analysis program (RAYBIO® ANALYSIS TOOL) provided by Raybiotech.

[b] The substantial signal was calculated only the signal intensity of positive value for the proteins whose secretions by hUCB-MSCs increased specifically after Aβ

treatment, rather than those protein secretions from hUCB-MSCs co-cultured with primary neuronal cells.

In order words, the signal intensity of pre-Aβ-treatment co-cultures condition (neuron + hUCB-MSCs) and the signal intensity indicating expression without hUCB-MSCs (neuron control, neuron + Aβ) were subtracted from the signal intensity of Aβ-treated co-cultures condition (neuron + Aβ + hUCB-MSCs).

When the signal intensity obtained from this calculation was above 0, the signal was referred to as a "substantial signal."

As a result, 19 proteins with substantial signals were selected. (The remaining proteins had signals below 0 and, thus were excluded). The proteins were those whose secretions increased specifically when hUCB-MSCs co-cultured with primary neuronal cells responded to Aβ treatment. In the manuscript, we described these proteins as being highly upregulate.

Human cytokine antibody array was used to screen for candidate proteins based on signal intensity values calculated according to the selection criteria.

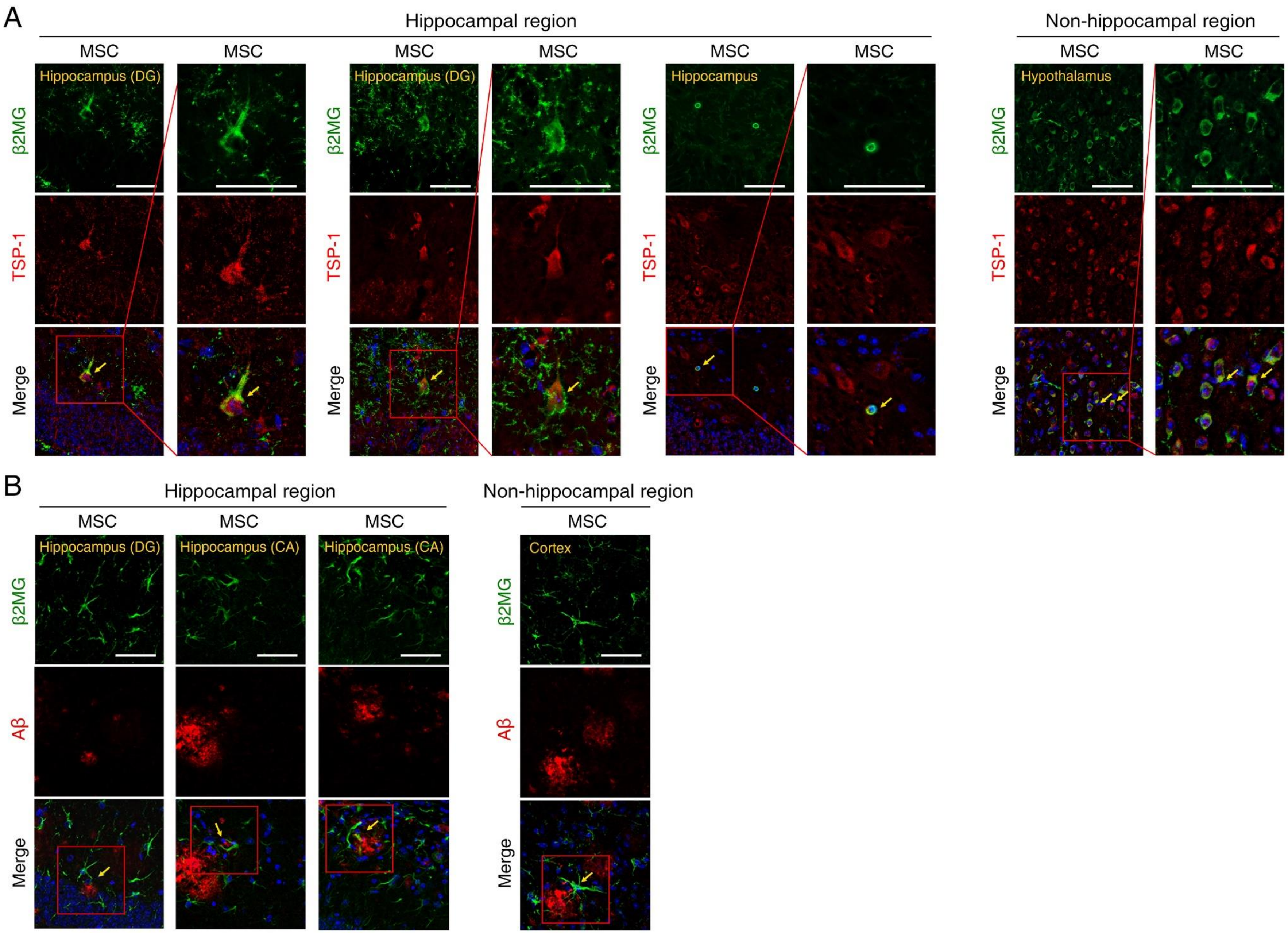
A
Hippocampal region
Non-hippocampal region
MSC
Hippocampus (DG)
Hippocampus
Hypothalamus
β2MG
TSP-1
Merge
B
Hippocampal region
Non-hippocampal region
MSC
Hippocampus (DG)
Hippocampus (CA)
Cortex
β2MG
Aβ
Merge

Supplementary Figure 1. TSP-1-secreted hUCB-MSCs can migrate to the regions of Aβ plaque in AD mouse brains. (**A**) Each tissue section of hippocampal and non-hippocampal regions was stained with DAPI, anti-β2MG, and anti-TSP-1 antibodies after the administration of hUCB-MSCs. The confocal images show merged green (β2MG, human-specific) and red (TSP-1) co-localisation in the hippocampal region and some non-hippocampal areas (Scale bars = 50 μm). Each boxed area was magnified and merged to analyse the co-localisation of β2MG-labelled hUCB-MSCs and TSP-1-secreting cells. (DG: dentate gyrus). (**B**) Each tissue section of hippocampal and non-hippocampal regions was stained with DAPI, anti-β2MG, and anti-Aβ antibodies after the administration of hUCB-MSCs. The confocal images show merged green (β2MG, human-specific) and red (Aβ) co-localisation in the hippocampal region and some non-hippocampal areas (Scale bars = 50 μm). Each boxed area indicates migrated hUCB-MSCs (β2MG-labelled) near Aβ deposits. (DG: Dentate Gyrus, CA: Cornu Ammonis).

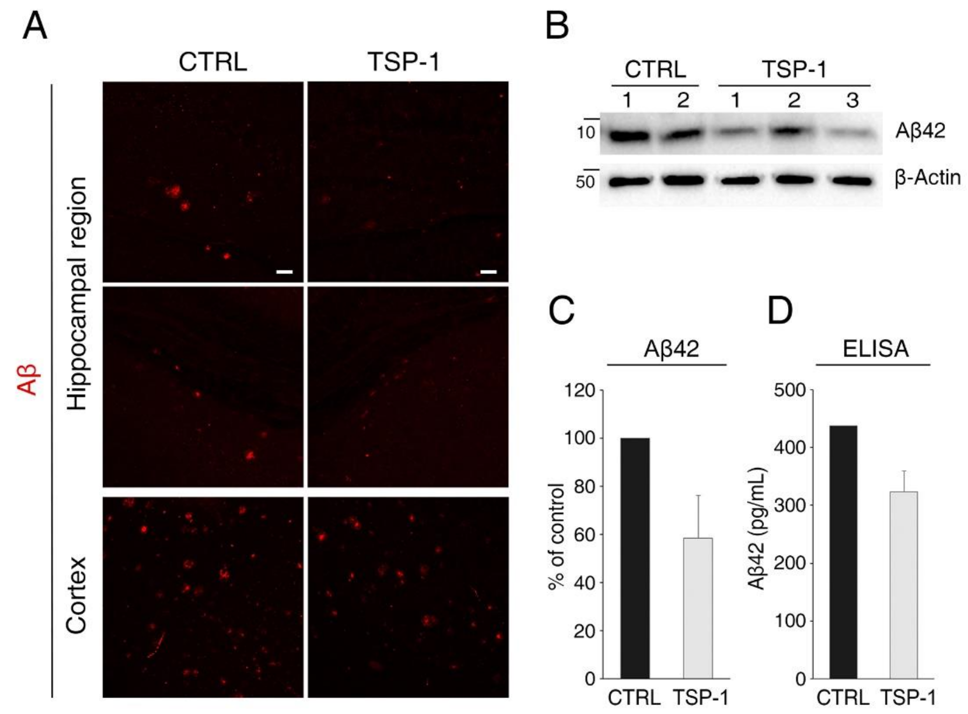
A
CTRL
TSP-1
Aβ
Hippocampal region
Cortex
B
CTRL
TSP-1
1
2
1
2
3
10
Aβ42
50
β-Actin
C
Aβ42
% of control
120
100
80
60
40
20
0
CTRL
TSP-1
D
ELISA
Aβ42 (pg/mL)
500
400
300
200
100
0
CTRL
TSP-1

Supplementary Figure 2. Injection of recombinant human TSP-1 into the brain parenchyma causes reduction of Aβ plaques. Recombinant human TSP-1 (1.0 μg/kg) was injected into the hippocampus of AD mouse brains (AP: -2.54, ML: ±3.0, DV: -2.5 mm, with reference to the bregma). (**A**) After 1 week, sections of the hippocampal regions and cortex were stained with specific antibodies for amyloid plaque (red). (**B**) The brains were then extracted and analysed for the level of Aβ42 with immunoblotting. β-Actin was used as a loading control. (n=2 for CTRL: 5XFAD control, n=3 for TSP-1: recombinant TSP-1-injected 5XFAD). (**C**) Immunoblotting was analysed by using densitometric quantification. (**D**) All brain tissue samples were evaluated by enzyme-linked immunosorbent assays.

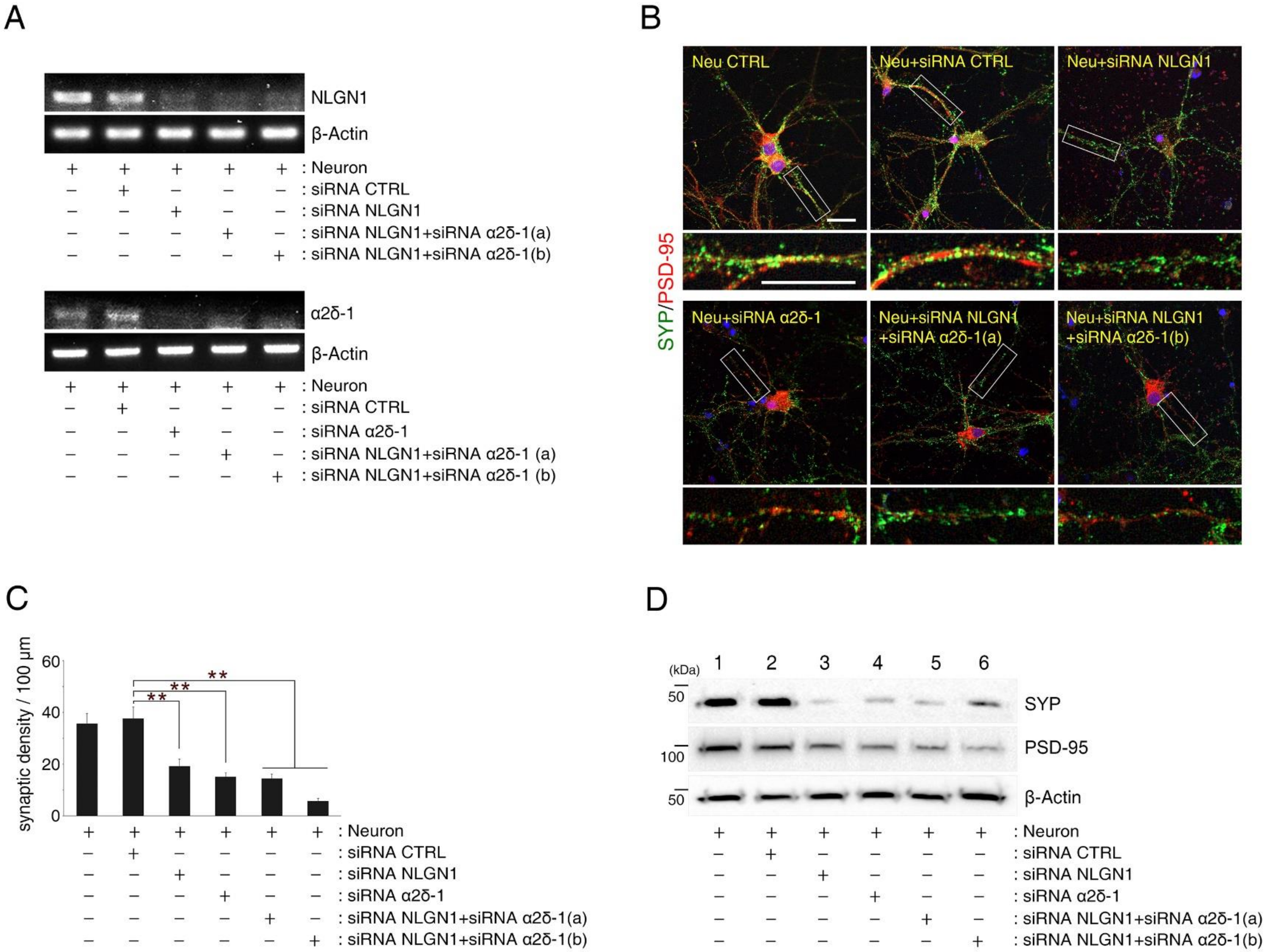
A
NLGN1
β-Actin
+ + + + + : Neuron
− + − − − : siRNA CTRL
− − + − − : siRNA NLGN1
− − − + − : siRNA NLGN1+siRNA α2δ-1(a)
− − − − + : siRNA NLGN1+siRNA α2δ-1(b)
α2δ-1
β-Actin
+ + + + + : Neuron
− + − − − : siRNA CTRL
− − + − − : siRNA α2δ-1
− − − + − : siRNA NLGN1+siRNA α2δ-1 (a)
− − − − + : siRNA NLGN1+siRNA α2δ-1 (b)
B
SYP/PSD-95
Neu CTRL
Neu+siRNA CTRL
Neu+siRNA NLGN1
Neu+siRNA α2δ-1
Neu+siRNA NLGN1 +siRNA α2δ-1(a)
Neu+siRNA NLGN1 +siRNA α2δ-1(b)
C
synaptic density / 100 µm
60
40
20
0
**
**
**
+ + + + + + : Neuron
− + − − − − : siRNA CTRL
− − + − − − : siRNA NLGN1
− − − + − − : siRNA α2δ-1
− − − − + − : siRNA NLGN1+siRNA α2δ-1(a)
− − − − − + : siRNA NLGN1+siRNA α2δ-1(b)
D
(kDa)
1 2 3 4 5 6
50
100
50
SYP
PSD-95
β-Actin
+ + + + + + : Neuron
− + − − − − : siRNA CTRL
− − + − − − : siRNA NLGN1
− − − + − − : siRNA α2δ-1
− − − − + − : siRNA NLGN1+siRNA α2δ-1(a)
− − − − − + : siRNA NLGN1+siRNA α2δ-1(b)

Supplementary Figure 3. Knockdown of TSP-1 receptors causes synaptic density loss in hippocampal neurons. (**A**) α2δ-1-siRNA or NLGN1-siRNA or both NLGN1-siRNA and α2δ-1-siRNA were transfected into hippocampal neurons overnight. Hippocampal neurons were separately transfected with scrambled siRNA as a control. mRNA expression of α2δ-1 or NLGN1 were analysed using RT-PCR. (**a**: NLGN1-siRNA and α2δ-1-siRNA, respectively; 12.5 nmol/L, **b**: NLGN1-siRNA and α2δ-1-siRNA, respectively; 25 nmol/L). (**B**) Representative images of hippocampal neurons stained for pre-synaptic (SYP, green) and post-synaptic (PSD-95, red) proteins (Scale bar = 25 μm). (**C**) Quantification of synaptic density (number of synapses per 100 μm of dendritic length, $n \geq 30$ dendrites) revealed that suppression of NLGN1, α2δ-1 and both NLGN1 and α2δ-1 by siRNA in the hippocampal neurons were induced synaptic by dysfunction in hippocampal neurons. (**a**: NLGN1-siRNA and α2δ-1-siRNA, respectively; 12.5 nmol/L, **b**: NLGN1-siRNA and α2δ-1-siRNA, respectively; 25 nmol/L). (** $p < 0.005$ versus control-siRNA-treated hippocampal neurons). (**D**) In NLGN1, α2δ-1, and both NLGN1 and α2δ-1-knockdown hippocampal neurons, SYP and PSD-95 expression levels were determined by immunoblotting under the same conditions in (**B, C**). Immunoblotting also showed remarkably attenuated expression of SYP and PSD-95 in α2δ-1-siRNA, NLGN1-siRNA, and both NLGN1-siRNA and α2δ-1-siRNA-treated hippocampal neurons.

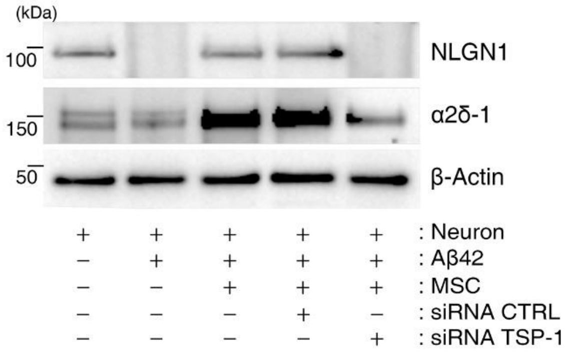

Supplementary Figure 4. Co-cultures with TSP-1 knockdown hUCB-MSCs decreases the expression of TSP-1 receptors in hippocampal neurons with Aβ peptide. NLGN1, and α2δ-1 expression levels were determined by immunoblotting under the same conditions in Fig. 3D. Immunoblotting showed attenuated expression of NLGN1 and α2δ-1 in co-culture with TSP-1-siRNA-treated hUCB-MSCs versus control-siRNA-treated hUCB-MSCs. β-Actin was used as a loading control.
